# Abnormal Crosstalk between Endothelial Cells and Podocytes Mediates Tyrosine Kinase Inhibitor (TKI)-Induced Nephrotoxicity

**DOI:** 10.3390/cells10040869

**Published:** 2021-04-12

**Authors:** Xiaoying Gu, Su Zhang, Ti Zhang

**Affiliations:** Department of Hepatobiliary Surgery, Tianjin Medical University Cancer Institute & Hospital, National Clinical Research Center for Cancer, Tianjin’s Clinical Research Center for Cancer, Key Laboratory of Cancer Prevention and Therapy, Tianjin 300060, China; guxiaoying@tmu.edu.cn (X.G.); zhangsu@tmu.edu.cn (S.Z.)

**Keywords:** angiogenesis, tyrosine kinase inhibitors, nephrotoxicity, endothelial cells, podocytes, crosstalk, Rac1, Cdc42

## Abstract

Vascular endothelial growth factor A (VEGFA) and its receptor VEGFR2 are the main targets of antiangiogenic therapies, and proteinuria is one of the common adverse events associated with the inhibition of the VEGFA/VEGFR2 pathway. The proteinuric kidney damage induced by VEGFR2 tyrosine kinase inhibitors (TKIs) is characterized by podocyte foot process effacement. TKI therapy promotes the formation of abnormal endothelial‒podocyte crosstalk, which plays a key role in TKI-induced podocyte injury and proteinuric nephropathy. This review article summarizes the underlying mechanism by which the abnormal endothelial‒podocyte crosstalk mediates podocyte injury and discusses the possible molecules and signal pathways involved in abnormal endothelial‒podocyte crosstalk. What is more, we highlight the molecules involved in podocyte injury and determine the essential roles of Rac1 and Cdc42; this provides evidence for exploring the abnormal endothelial‒podocyte crosstalk in TKI-induced nephrotoxicity.

## 1. Introduction

Vascular endothelial growth factor A (VEGFA) and its receptor VEGFR2 are the main drivers of tumor angiogenesis and the main targets of antiangiogenic therapies [1]. VEGFA‒VEGFR2 inhibitors can be classified into two categories based on their targets: drugs that directly inhibit VEGFA, such as bevacizumab and VEGFA trap versus tyrosine kinase inhibitors (TKIs) that inhibit VEGFR2, such as sorafenib, lenvatinib, apatinib, regorafenib [2], etc. Inhibition of the VEGFA/VEGFR2 pathway has shown remarkable efficacy in improving the prognosis of patients with cancer. At the same time, these drugs also lead to some unignorable adverse events such as proteinuria, hypertension, hand‒foot syndrome, etc. Among these adverse events, proteinuria attracts attention for its high incidence, great impact on cancer treatment, and lack of effective interventions. In two phase II clinical trials published recently, the incidence of proteinuria in patients taking regorafenib and lenvatinib was 58% [3] and 83% [4], respectively. In patients taking lenvatinib, proteinuria was the most frequent adverse event contributing to dose reduction; it resulted in 52% of participants experiencing dose reduction [4]. When the VEGFR2 monoclonal antibody ramucirumab was combined with other types of TKI, such as the EGFR inhibitor erlotinib, the incidence of proteinuria was significantly increased (ramucirumab vs. ramucirumab + erlotinib: 20% vs. 34%), and proteinuria was the most common adverse event that led to ramucirumab dose reduction [5]. The administration of other VEGFA‒VEGFR2 inhibitors also caused proteinuria (Table 1) [6,7,8,9,10,11]. A reduction in the dose of these agents inevitably affects their antitumor effect. Despite the fact that angiotensin II receptor blockade (ARB) effectively reduced proteinuria in various kidney diseases such as diabetic nephropathy, obesity-induced renal disease, and HIV-associated nephropathy [12,13,14,15], and that angiotensin-converting enzyme inhibitors (ACEIs) or ARB are recommended for alleviating proteinuria caused by diabetic nephropathy according to guidelines for treating diabetic nephropathy, in clinical practice, ARB did not effectively alleviate the proteinuria caused by bevacizumab; the patient had persistently elevated proteinuria while taking telmisartan [16]. The administration of ACEIs even compromised the anticancer efficacy of antiangiogenic drugs [17,18]. These facts make proteinuria an urgent problem, and the key to solving this problem is to thoroughly understand the mechanism of proteinuria induced by VEGFA‒VEGFR2 inhibitors.

Under physiological conditions, except for mediating angiogenesis, the VEGFA/VEGFR2 pathway also plays an important role in the development and function maintenance of the kidneys [24]; the inhibition of different molecules on this pathway leads to different kidney pathological phenotypes. VEGFA inhibitors mainly resulted in renal thrombotic microangiopathy (TMA) with glomerular endothelial swelling and focal glomerular capillary thrombosis as the main pathological features [25], while renal biopsies from patients accepting TKI therapy showed minimal change nephropathy/focal segmental glomerulopathy (MCN/FSG)-like lesions, characterized by podocyte foot process effacement [26,27,28]. However, considering the small number of patients enrolled in these studies, this conclusion needs further verification. In addition to TKIs, there are various etiologies leading to proteinuric nephropathies characterized by MCN/focal segmental glomerulosclerosis (FSGS), such as diabetic nephropathy. Despite the similar pathological types, TKI-induced nephropathy is quite different from the others. For example, the expression levels of VEGFA and VEGFR2 were significantly increased in early diabetic nephropathy, the inhibition of the VEGFA/VEGFR2 pathway attenuated proteinuria [29], while in patients taking TKIs, the expression levels of VEGFA and VEGFR2 were lower than normal [17,28], which is the opposite of diabetic nephropathy. This indicated that, even with similar pathological changes, the mechanism may be totally different.

Proteinuria is closely related to the destruction of integrity of the glomerular filtration barrier, which is composed of podocytes, a glomerular basement membrane, and endothelial cells [30]. Highly differentiated, specialized podocytes are essential to maintain the integrity of the glomerular filtration barrier [30]. Podocytes have a complex structure consisting of three subcellular compartments: a cell body, microtubule-driven primary processes, and actin-driven foot processes [31]. Actin cytoskeletal remodeling results in podocyte foot process effacement, which is the main structural change leading to proteinuria [30,32]. In normotensive and hypertensive rats, VEGFR-2 kinase inhibition treatment resulted in podocyte foot process effacement [33], and the specific mechanism remains unclear. This review mainly focuses on TKI-induced proteinuric nephropathy and summarizes its possible underlying mechanisms.

## 2. VEGFA/VEGFR2 Pathway and Proteinuria

Podocyte injury is thought to be the initiating cause of proteinuric kidney diseases [34]. However, the comparison of kidney damage associated with TKIs and VEGFA inhibitors demonstrated that the former was characterized by high expression of c-maf-inducible protein (c-mip) in podocytes, while the latter was characterized by significantly increased expression levels of RelA (also called p65 NF-κB) in podocytes and endothelial cells [28]. Under normal conditions, RelA was only expressed in podocytes and prevented the transcriptional activation of c-mip by binding to its promoter [28]. This indicated that the glomerulopathy associated with TKIs mainly affected podocytes, whereas anti-VEGFA therapy mainly affected endothelial cells and podocyte injury may be a secondary event [28]. Correspondingly, in a podocyte-specific VEGFA deletion experimental murine model, endothelial damage and proteinuria occurred prior to podocyte injury after eliminating VEGFA production from podocytes [25].

Coincidentally, in the process of transforming growth factor-β (TGF-β) inducing FSGS, endothelial damage and proteinuria caused by the overexpression of podocyte-specific TGF-β occurred before podocyte injury [35]. This study demonstrated the role of abnormal crosstalk between endothelial cells and podocytes in kidney injury. Podocyte-specific TGF-β signaling promoted the release of endothelin-1 (EDN1) from podocytes, thereby activating EDN1 receptor type A (EDNRA) on the surface of endothelial cells, which mediated mitochondrial oxidative stress and the dysfunction of adjacent endothelial cells. Subsequently, endothelial dysfunction promoted proteinuria, podocyte apoptosis, glomerulosclerosis, and renal failure, which can be prevented by inhibiting EDNRA or scavenging mitochondrial-targeted ROS [35]. A similar sequence of cell damage suggests the existence of abnormal crosstalk between podocytes and endothelial cells after anti-VEGFA treatment.

For patients treated with TKIs or VEGFA inhibitors, no one developed MCN/FSG-like lesions and TMA at the same time [28], but both lesions were observed in different patients receiving the same treatment regimen [26,28]. Our previous study achieved the same results: both TMA and FSG-like lesions were observed in an apatinib-induced proteinuria mouse model [17]. In normal kidneys, VEGFA is mainly produced by podocytes and binds to VEGFR2 on the surface of endothelial cells [36] to maintain the structure and function of the endothelium. TKIs and VEGFA inhibitors all function by blocking the VEGFA/VEGFR2 pathway; the related kidney injury is not only associated with endothelial damage, but also with podocyte injury. These findings further indicate that VEGFA‒VEGFR2 inhibitors contribute to kidney injury by mediating the formation of abnormal crosstalk between podocytes and endothelial cells, rather than only interrupting the VEGFA/VEGFR2 signaling pathway.

## 3. The Mechanism Underlying Abnormal Endothelial‒Podocyte Crosstalk

Although VEGFA‒VEGFR2 inhibitors reduce the levels of VEGFA and VEGFR2 in the kidneys of patients with cancer [17,28], not all patients develop proteinuria. Despite the fact that VEGFA and VEGFR2 are the main drivers of angiogenesis, there are many other pro-angiogenic factors [37,38], some of which have been found to be associated with kidney injury. For example, the overexpression of TGF-β caused proteinuria [35], while the increased expression of angiopoietin-like factor 4 (Angptl4) reduced proteinuria by interacting with glomerular endothelial αvβ5 integrin [39].

These findings provide a possible explanation for the underlying mechanism by which VEGFA‒VEGFR2 inhibitors mediate kidney injury and proteinuria through intercellular crosstalk: the administration of VEGFA‒VEGFR2 inhibitors induces glomerular capillary endothelial damage by blocking the normal crosstalk mediated by VEGFA/VEGFR2 between podocytes and endothelial cells. In the early stage of TKI administration, the degree of endothelial dysfunction is mild and not enough to produce proteinuria. In patients without proteinuria, the damaged endothelial cells may promote the compensatory production of nephroprotective pro-angiogenic factors. On the contrary, in kidneys of patients with proteinuria, the abnormal endothelial‒podocyte crosstalk can lead to podocyte injury through directly acting on podocytes or inducing the production of compensatory pro-angiogenic factors, which are harmful to podocytes. As kidney damage gradually worsens, endothelial cells can be affected. Upon the administration of VEGFA inhibitors, serious endothelial damage can directly lead to proteinuria and then cause podocyte injury through the same process as TKI administration. There are no studies on this hypothesis so far, which requires further exploration.

## 4. Possible Endothelial‒Podocyte Crosstalk in TKI-Induced Proteinuria

Current studies on other kidney diseases elucidate several molecules and signal pathways mediating endothelial‒podocyte crosstalk and participating in the development of proteinuric nephropathies. These molecules and related signaling pathways may be involved in the occurrence of TKI-induced proteinuric nephropathy.

### 4.1. Neuropilin-1 (NRP1)

In the regulation of VEGF signaling, NRP1 plays an important role in endothelial cells. NRP1 is a co-receptor of receptor tyrosine kinases that can enhance the activity of VEGFR2 by binding to VEGFR2 in a VEGFA-dependent manner and can increase the motility of endothelial cells [40]. The complex between NRP-1 and VEGFR2 can be formed between receptors present on different cells [40], making it possible for NRP-1 to mediate endothelial‒podocyte crosstalk. In the glomeruli of diabetic nephropathy mice, the expression of the NRP-1 protein was reduced in podocytes, and the reversals of podocyte injury and proteinuria were associated with the restoration of NRP-1 expression [41]. The alteration of NRP1 expression in the kidneys during TKI therapy and whether NRP1 is involved in the development of podocyte injury and proteinuria mediated by TKIs remain unclear.

### 4.2. Angiopoietin1 (ANGPT1)/TIE2 Pathway

In the kidneys, ANGPT1 is mainly produced by podocytes and acts through the TIE2 expressed by endothelial cells. In the process of renal blood vessel development, this signaling pathway can promote blood vessel maturation [36]. In inflammation, ANGPT1 can stabilize vascular endothelial‒cadherin (VE‒cadherin) at the adherens junctions by activating Rac1, which enhances vascular barrier defense against inflammatory stimuli; additionally, it can reduce the infiltration of immune cells. All these contribute to maintaining the stabilization of the endothelium [42]. While in developed kidney, most research on ANGPT1/TIE2 has focused on its role in kidney disease. In the early stage of diabetic kidney disease, the expression of ANGPT1 in the kidneys was upregulated and returned to the control level or lower in the later stage [36]; podocyte-specific inducible repletion of ANGPT1 in early diabetic kidney disease reduced albuminuria [43], indicating a protective role of ANGPT1 for the kidneys, which may be attributed to the anti-inflammatory effect and the maintenance of vascular integrity mediated by ANGPT1. In tumor therapy, TIE2 is the target of some TKIs [19,44]. In brain tumors, VEGFR2 inhibition promoted vascular normalization through the upregulation of ANGPT1, enhancing the outcome of combined radiation therapy [45]. The combination of exogenous ANGPT1, TKIs, and other antitumor drugs may alleviate proteinuria while enhancing the antitumor effect.

### 4.3. Autocrine Epidermal Growth Factor (EGF)/EGF Receptor (EGFR)

Previous studies have demonstrated the role of EGF/EGFR in various nephropathies. In rapidly progressive crescentic glomerulonephritis, the expression of heparin-binding EGF (HB-EGF) was found to be significantly elevated [46]. In vitro, podocytes from Hbegf (−/−) glomeruli presented a migratory phenotype. The administration of EGFR-TKI or podocyte-specific EGF knockout prevented the increase in podocyte motility [46]. In Hbegf (+/+) mice, the induction of nephrotoxic serum (NTS) resulted in mild to severe podocyte foot process effacement, while the administration of EGFR-TKI or podocyte-specific EGF knockout improved proteinuria, podocyte injury, kidney damage and decreased renal function [46]. In podocytes of diabetic nephropathy, downregulated Gprc5a mediates podocyte foot process effacement, FSGS, and proteinuria by upregulating EGFR and TGF-β signaling pathways [47]; an upregulated EGFR signal can also promote the activation of the TGF-β signaling pathway [48]. Additionally, EGFR can be transactivated. Agonists such as angiotensin II, endothelin, IL-8, ROS, TGF-β1, combined with G-protein coupled receptors to activate intracellular kinases such as Src and PKC, subsequently cleaved EGFR ligands through phosphorylating A disintegrin and metalloproteinase (ADAM) family members [36,49]. The transactivation of EGFR increases the possibility of EGF/EGFR being involved in the nephropathy related to TKIs, since the metabolites of the damaged endothelial cells may lead to podocyte injury through this way. However, although EGFR-TKI alleviated proteinuria and kidney injury in crescentic glomerulonephritis [46], in clinical trials, the combination of VEGFR2 monoclonal antibody ramucirumab and EGFR inhibitor erlotinib resulted in the increased incidence of proteinuria compared to ramucirumab alone [5]. Therefore, we should be cautious in using EGFR-TKI to alleviate the proteinuria induced by VEGFR2 inhibition, and further research is required to clarify the change in the EGF/EGFR signaling pathway during TKI therapy.

### 4.4. Semaphorin3A (Sema3A)

Sema3A is mainly produced by mature podocytes and is thought to be involved in endothelial‒podocyte crosstalk in diabetic nephropathy [29]. Previous studies reported that this molecule seems to mediate podocyte injury and proteinuria through the downregulation of VEGFA/VEGFR2 signaling [29,36]. After the injection of exogenous Sema3A, the kidney showed podocyte foot process effacement and endothelial damage, accompanied by the decreased expression of VEGFR2 and the downregulation of podocin, nephrin, and CD2-related proteins (CD2-AP), all of which were prevented by the co-administration of VEGF165 and Sema3A [50]. This demonstrated that the change in the expression of Sema3A may not be the reason for VEGFA‒VEGFR2 inhibitor-related proteinuria; it is more likely a susceptibility factor—that is, patients having a higher level of Sema3A at the onset of taking VEGFA‒VEGFR2 inhibitors may be prone to developing proteinuria. However, the role of Sema3A in tumors is complex. In mouse models of pancreatic neuroendocrine tumors and cervical carcinoma, the expression of Sema3A overcomes proinvasive and prometastatic resistance [51], while the knockdown of Sema3A increased the sensitivity of Lewis lung cancer cells to EGFR-TKIs gefitinib and erlotinib [52]. Therefore, alleviating TKI-induced proteinuria without affecting the anticancer effect of TKIs through targeting Sema3A is a huge challenge.

### 4.5. CXCL12/CXCR4

Chemokine CXCL12 (SDF-1) and its receptor CXCR4 are expressed by podocytes and endothelial cells, respectively [36]. This system is vital to glomerular vascular development. CXCL12- and CXCR4-deficient kidneys had identical abnormal blood vessel development that featured the ballooning of the developing glomerular tuft and disorganization of the renal vasculature [53]. In the process of neo-angiogenesis, CXCL12 plays an essential role in the recruitment of CXCR4 (+) pro-angiogenic bone marrow (BM) cells, including subsets of hematopoietic cells and endothelial progenitor cells [54], and this system can work synergistically with the VEGF system, the latter subsequently promoting arterial differentiation [55]. However, the function of CXCL12/CXCR4 in podocyte injury and kidney diseases is controversial. The inhibition of CXCL12 in mice with diabetic nephropathy alleviated glomerulosclerosis, prevented proteinuria, and podocyte loss [56], but in another study, dipeptidyl peptidase-4 (DPP-4) inhibition suppressed the progression of diabetic nephropathy by upregulating CXCL12, indicating a protective effect of CXCL12 on podocytes and kidneys [57]. In VEGFR-expressing glioblastoma, it has been confirmed that VEGFR inhibitors upregulated CXCR4 through TGF-β/TGF-β receptor [58], and the inhibition of CXCR4 enhanced the efficacy of sunitinib (a multitarget TKI) in preclinical models of human glioblastoma [59]. In TKI-induced proteinuric kidney disease, the expression of the CXCL12/CXCR4 system may be altered to participate in the abnormal endothelial‒podocyte crosstalk or to protect podocytes and maintain the integrity of the vascular system. More investigations are required to determine the specific role of the CXCL12/CXCR4 system in TKI-induced proteinuria.

### 4.6. WT1

WT1 is a podocyte-specific transcription factor that can promote normal crosstalk between podocytes and endothelial cells by modulating the bioavailability of VEGFA. In normal kidneys, WT1 promoted the expression of 6-O-endosulfatases (Sulf) to reduce the 6-O-sulfation of heparan sulfate chains within heparan sulfate proteoglycans (HSPGs) on the surface of podocytes, inhibiting the binding of HSPGs and VEGFA, and increased the release of VEGFA by podocytes [60]. WT1 can also directly upregulate the expression of VEGFA [61]. In the podocytes of FSGS, elevated expression of microRNA-193a reduced WT1 expression and thereby downregulated target genes of WT1 that are vital to podocyte structure, including *PODXL* (podocalyxin), *NPHS1* (nephrin), *VEGFA* (VEGFA), etc., eventually leading to kidney disease and proteinuria [61]. Based on these results, the kidneys of patients with lower WT1 expression at the onset of administration of VEGFA‒VEGFR2 inhibitors may be more vulnerable to these agents.

### 4.7. Endothelial Thrombomodulin-Protein C

Recent studies have found that the coagulation system participates in the development of kidney disease through its non-hemostatic function. Endothelial‒podocyte crosstalk mediated by the thrombomodulin‒protein C system was found in diabetic nephropathy [62]. Thrombomodulin expressed in glomerular endothelial cells activated endothelial protein C by binding to thrombin, and then activated protein C (aPC) promoted the dimerization of PAR3 and PAR2 in human podocytes and that of PAR3 and PAR1 in murine podocytes through activating PAR3, which subsequently limited the production of mitochondrial ROS by suppressing the redox enzyme p66Shc [62]. This crosstalk between endothelial cells and podocytes can protect podocytes from injury, which has been confirmed by the improvement of diabetic nephropathy in mice through exogenous administration of aPC [63]. Inflammation or cell dysfunction can reduce the expression of thrombomodulin and destroy this protective crosstalk [62]. Currently, protective effects of aPC on endothelial cells and podocytes, as well as the improvement of vascular damage, were found in the case of high VEGF expression [63,64,65]. Further verification is needed to explore the possibility that the damage of endothelial cells mediates podocyte injury and proteinuria through the destruction of the thrombomodulin‒aPC pathway in the early stage of TKI therapy.

### 4.8. Integrin-Linked Kinase (ILK)

ILK is a serine threonine kinase that can interact with the cytoplasmic domain of β-integrin, playing a key role in regulating integrin-mediated podocyte‒matrix adhesion [66,67]. Under physiological conditions, the complex formed by ILK, nephrin, and α-actinin-4 maintains the normal foot process structure of the podocytes and the integrity of the slit diaphragm. The podocyte-specific ablation of ILK destructed this complex, leading to severe proteinuria, glomerulosclerosis, and renal failure [68]. In podocyte injury and proteinuria caused by various injury stimuli, ILK expression was increased in podocytes. The blockade of ILK activity maintained the normal shape and function of podocytes [69]. ILK is the target gene of TGF-β/Smad signaling [66]. TGF-β has been confirmed to play a central role in the phenotypic transition of renal tubular epithelial cells and podocytes after injury [66]. In response to hypoxia or oxidative stress, renal tubular epithelial cells mediate the infiltration of inflammatory cells by producing various chemokines and cytokines. The mixture of pro-inflammatory cytokines produced by these inflammatory cells can enhance TGF-β signaling by inducing the expression of TGF-β receptors, and TGF-β can be produced by injured tubular epithelial cells or inflammatory cells [66]. TKI therapy can induce oxidative stress [70,71,72] and has been confirmed to induce upregulated TGF-β [73,74], which may mediate podocyte damage in a way similar to that of the tubular epithelial cell injury induced by TGF-β. Additionally, ILK may also be associated with TKIs through c-mip. In membranous nephropathy, the upregulation of c-mip was closely related to podocyte dysfunction, as well as the downregulation of synaptopodin and upregulation of ILK [75]. Additionally, sorafenib has been proven to induce the high expression of c-mip in podocytes [28]. However, the changes in ILK in kidneys treated with TKIs have not yet been reported yet. In tumor cells, the high expression of ILK can promote tumor angiogenesis and tumor progression by inducing VEGF expression [76,77,78]. Highly expressed ILK was an independent poor prognostic factor for progression-free survival in patients with non-small-cell lung cancer (NSCLC) [79], while the knockdown of ILK gene made lung squamous cell carcinoma sensitive to TKI therapy [80]. In prostate cancer, the anticancer effect of TKI therapy was related to the reduced expression of ILK [81]. Together, these factors demonstrate that ILK may be a potential target to simultaneously reduce proteinuria and enhance the anticancer efficacy of TKIs.

### 4.9. Oxidative Stress

In the human body, the oxygen consumption and mitochondrial enrichment of the kidney is second only to the heart [82]. Studies have emphasized the involvement of mitochondria in the process of chronic kidney injury [83,84], which is mainly due to the reduction in mitochondrial DNA replication, loss of mitochondrial membrane potential, and decreased ATP production [85]. In chronic kidney disease, the progressive deterioration of renal function can induce some biological dysfunction, including changes in cellular energy metabolism, protein malnutrition, and the synthesis of a large number of oxidative stress mediators [86,87]. Some studies have reported that, even in the very early stages of chronic kidney disease, there is a large amount of reactive oxygen generated and upregulation of oxidative stress markers, such as lipid peroxide (MDA). The levels of all these oxidative stress markers are negatively correlated with the glomerular filtration rate [88,89,90,91]. As mentioned above, the mitochondrial dysfunction induced by EDN1/EDN1 receptor in glomerular endothelial cells can lead to podocyte loss, proteinuria, and glomerular sclerosis, which can be prevented by inhibiting EDN1 receptor or mitochondrial-mediated oxidative stress [35]. The same results were verified in animal models of diabetic nephropathy, suggesting the existence of crosstalk between glomerular endothelial injury and podocytes [92]. In the development of proteinuria induced by sunitinib, the EDN1/EDN1 receptor pathway and oxidative stress have been confirmed to play important roles [71]. These factors indicate the possibility that TKIs may induce podocyte injury and proteinuria through the abnormal endothelial‒podocyte crosstalk mediated by oxidative stress.

Taken together, these molecules and related pathways have the ability to participate in the abnormal endothelial‒podocyte crosstalk mediating TKI-induced proteinuric kidney disease, but their specific role needs further exploration.

## 5. Cell Motility-Related Molecules: Rac1 and Cdc42

According to the conjecture above, podocyte injury is the key to TKI-induced nephropathy, and endothelial damage is the initial factor. Podocyte injury can, therefore, be taken as the entry point to find the compensatory pro-angiogenic factors. In the process of angiogenesis, vascular barrier repair, and podocyte foot process effacement, both endothelial cells [93] and podocytes [94] show increased motility. This similarity provides evidence for finding the compensatory pro-angiogenic factors that participate in abnormal endothelial‒podocyte crosstalk in the proteinuric nephropathy associated with TKIs.

Endothelial cell migration is a key step of angiogenesis mediated by pro-angiogenic factors [93]; blocking this step significantly inhibits angiogenesis [95,96]. The actin cytoskeleton plays a crucial role in endothelial cell migration through continuous remodeling into filopodia, lamellipodia, and stress fibers [93]. The formation of filopodia is mainly regulated by the activation of Rho GTPase family member Cdc42, and the formation of lamellipodia is closely related to actin polymerization involving Rac1, another member of the Rho family GTPases [93].

In podocyte damage and proteinuria, cytoskeletal regulation also plays an important role. The summarization of molecules involved in the regulation of the podocyte actin cytoskeleton and their signaling pathways indicates that Rac1 and Cdc42 may be the key intersections mediating angiogenesis and podocyte injury induced by the compensatory pro-angiogenic factors mentioned above.

### 5.1. The Role of Rac1 and Cdc42 in Podocyte Injury

The Rho GTPase family members involved in podocyte foot process effacement and proteinuric nephropathies include RhoA, Cdc42, and Rac1. The activation of RhoA mediates the formation of stress fibers in podocytes and the maintenance of the integrity of podocyte actin cytoskeleton [97], keeping podocytes in a healthy stationary phenotype [94]; on the contrary, the activation of Cdc42 and Rac1 leads to podocyte injury and proteinuria [98,99,100].

**Figure 1:**
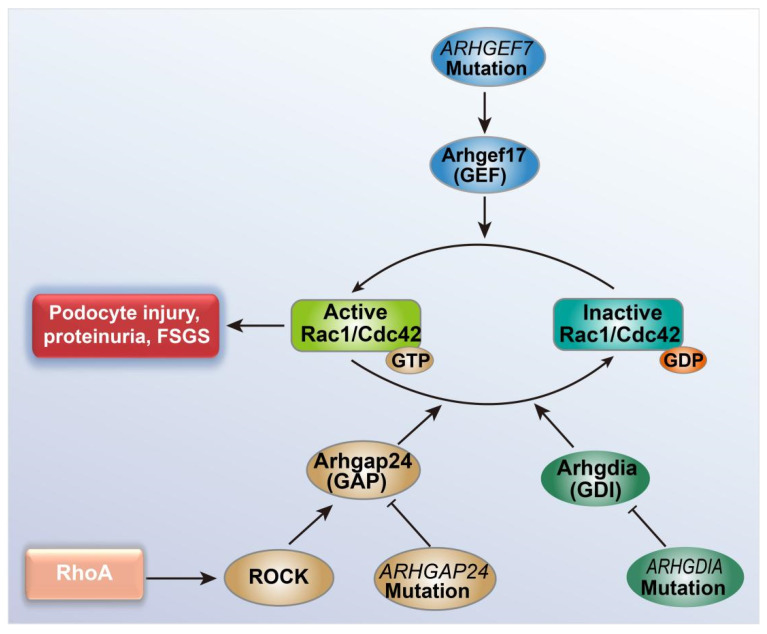
The relationship between FSGS and Rac1/Cdc42 activity transition. Rac1/Cdc42 changes from the GDP binding-inactive state to the GTP binding-activated state, which can cause FSGS characterized by podocyte damage and proteinuria. RhoA can activate GTPase activating protein Arhgap24 through its effector Rho kinase ROCK to inhibit this conversion. The *ARHGAP24*, *ARHGDIA*, and *ARHGEF17* gene mutations in FSGS can increase the activity of Rac1/Cdc42 by inhibiting GAP activity and promoting GEF activity, respectively. Abbreviations: FSGS, focal segmental glomerulosclerosis; Arhgap24, Rho GTPase activating protein 24; GAP, GTPase activating protein; ROCK, Rho-associated kinase; Arhgdia, Rho GDP-dissociation inhibitor; GDI, GDP-dissociation inhibitor; Arhgef17, Rho Guanine Nucleotide Exchange Factor17; GEF, guanine nucleotide exchange factor.

The mutual antagonism between RhoA, Rac1, and Cdc42 depends on RhoA inhibiting the activities of Rac1 and Cdc42. The activities of Rho family GTPases are regulated by guanine nucleotide exchange factors (GEFs), GTPase-activating proteins (GAPs), and GDP dissociation inhibitors (GDIs), among which the GEFs activate Rho GTPases from an inactive GDP-bound state to an active GTP-bound state, whereas GAPs inactivate Rho GTPases and GDIs inhibit Rho GTPases activities (Figure 1) [94]. RhoA can activate Arhgap24 protein (GAP) through its effector kinase ROCK to inactivate Rac1 [101]. The gene encoding Arhgap24 protein mutated in FSGS, and this mutation reduced the ability of Arhgap24 protein to convert Rac1 to an inactive state. The increased Rac1 activity in podocytes resulted in altered podocyte cell shape and membrane dynamics, and ultimately led to podocyte injury and proteinuria [102]. Additionally, *ARHGDIA* mutations [103] and *ARHGEF17* mutations [104] (the genes encoding GDI and GEF, respectively) were also found in FSGS, both of which were closely relevant to the pathogenesis of FSGS (Figure 1), confirming that increased Rac1 activity was essential for podocyte injury and proteinuria.

Excluding the fact that mutations in genes encoding regulatory proteins increased the activity of Rac1 and Cdc42, other factors also triggered altered activities of these two molecules when mediating podocyte injury and proteinuria.

### 5.2. Podocyte Injury and Transient Receptor Potential (TRP) Channels

Actin cytoskeletal remodeling is a calcium ion-dependent process; transient receptor potential (TRP) channels can generate calcium ion microdomains in many cell types and therefore participate in this process [105]. Among the TRPCs, TRPC5 and TRPC6 are the ones commonly studied in podocyte injury and Rho family GTPases are closely involved in TRPC-mediated podocyte injury. The calcium influx mediated by the activation of TRPC5 and TRPC6 in podocytes can antagonistically regulate actin dynamics and cell motility by stimulating the activation of Rac1 and RhoA, respectively [105]. The balance of calcium signals through TRPC5 and TRPC6 seems to be important to normal podocyte structure and function. Under physiologic conditions, the activation of TRPC6 is greater than that of TRPC5. Excessive activation of both TRPC6 and TRPC5 leads to podocyte injury and proteinuria [106]. TRPC6 expression has been found to be increased in patients with several acquired nephropathies [107]. TRPC5 can lead to podocyte injury and proteinuria through interaction with Rac1. The activation of Rac1 promoted the insertion of receptor-activated TRPC5 into the plasma membrane of podocytes [105,108]. Under the activation of angiotensin II type 1 receptor (AT1R), calcium influx mediated by TRPC5 further stimulated Rac1 activation, resulting in a positive feedback that led to podocyte actin cytoskeletal remodeling and foot process effacement. Both TRPC5 inhibitor AC1903 and Rac1 inhibitor alleviated the podocyte apoptosis and proteinuria induced by constitutively activated AT1R in podocytes [109,110]. However, a recent study found that TRPC5 was not involved in the occurrence or aggravation of kidney disease [111]. These two studies had completely opposite results. A discussion proposed that TRPC5 mutation has not been identified in human kidney diseases, which means the role of TRPC5 in podocyte injury and kidney disease remains to be clarified [112].

### 5.3. Podocyte Injury and Synaptopodin

**Figure 2:**
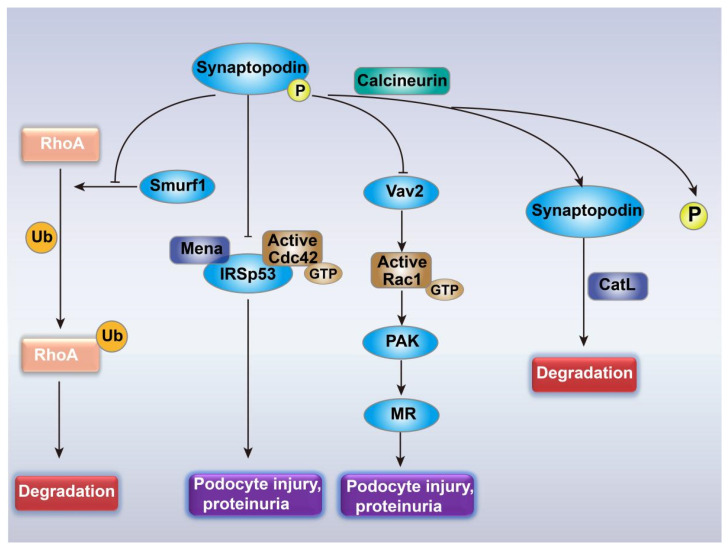
The connection between proteinuria and Rho GTPase family. Abbreviations: CatL, cathepsin L; Mena, an Ena/VASP family protein; IRSp53, insulin receptor substrate 53; Smurf1, smad ubiquitination regulatory factor 1; Vav2, a guanine nucleotide exchange factor; PAK, p21-activated kinase; MR, mineralocorticoid receptor. P, phosphorylation; Ub, ubiquitination.

Synaptopodin plays an important role in the maintenance of the integrity of the podocyte actin cytoskeleton. In this process, synaptopodin is closely associated with the Rho family GTPases (Figure 2). It can competitively inhibit Smurf1-mediated RhoA ubiquitination, preventing it from degradation by proteasomes [97]. Synaptopodin can also alleviate podocyte injury and proteinuria caused by the Cdc42:IRSp53: Mena signaling complexes by competitively binding to insulin receptor substrate 53 (IRSp53) and blocking the binding of Cdc42 and Mena to IRSp53 [98]. Additionally, synaptopodin can inhibit Rac1 activity by inhibiting the activation of Vav2 (GEF) [100]. The calcium ion-dependent calcineurin can mediate the dephosphorylation of synaptopodin, thereby destroying the phosphorylation-dependent interaction between synaptopodin and 14-3-3β, making synaptopodin suffer from cathepsin L (CatL)-mediated degradation [113]. Cyclosporine A (CsA) maintained the stability of the podocyte actin cytoskeleton by blocking the effect of calcineurin on synaptopodin [113], and alleviated the proteinuria caused by FSGS [114]. The relationship between synaptopodin and the Rho family GTPases suggests that patients may gain a similar benefit as from CsA—reducing proteinuria due to the inhibition of Rac1 and Cdc42 activity—but without suffering from the side effects of CsA.

### 5.4. Mineralocorticoid Receptor (MR)-Mediated Podocyte Injury

Many drugs reduce proteinuria by inhibiting the renin‒angiotensin‒aldosterone system (RAAS) [12,13,115]; mineralocorticoid receptor (MR) antagonists Esaxerenone and Finerenone have also shown good effects in reducing proteinuria in clinical trials [116,117]. However, aldosterone is not the only stimulus that increases the expression of MR in patients with proteinuria; factors outside the RAAS system can also mediate the increased expression and activation of MR. A previous study quantified the serum aldosterone and mRNA expression level of MR in renal biopsies from 95 patients with mild to heavy proteinuria and found that serum aldosterone level was not related to MR mRNA expression or proteinuria; the mRNA expression of MR significantly increased in patients with heavy proteinuria when compared with patients with no proteinuria or mild proteinuria [118]. Further studies found that Rac1 promoted the expression of MR mRNA and protein in an aldosterone-independent manner and facilitated MR nuclear translocation and activation through p21-activated kinase (PAK) phosphorylation, which in turn caused podocyte damage and proteinuria (Figure 2) [99]. The administration of a Rac1 inhibitor significantly reduced the enhanced MR signal transduction in kidneys of Arhgdia-/-mice and alleviated proteinuria [99].

### 5.5. Podocyte Damage and Proteinuria Mediated by the uPAR‒αvβ3 Pathway

**Figure 3:**
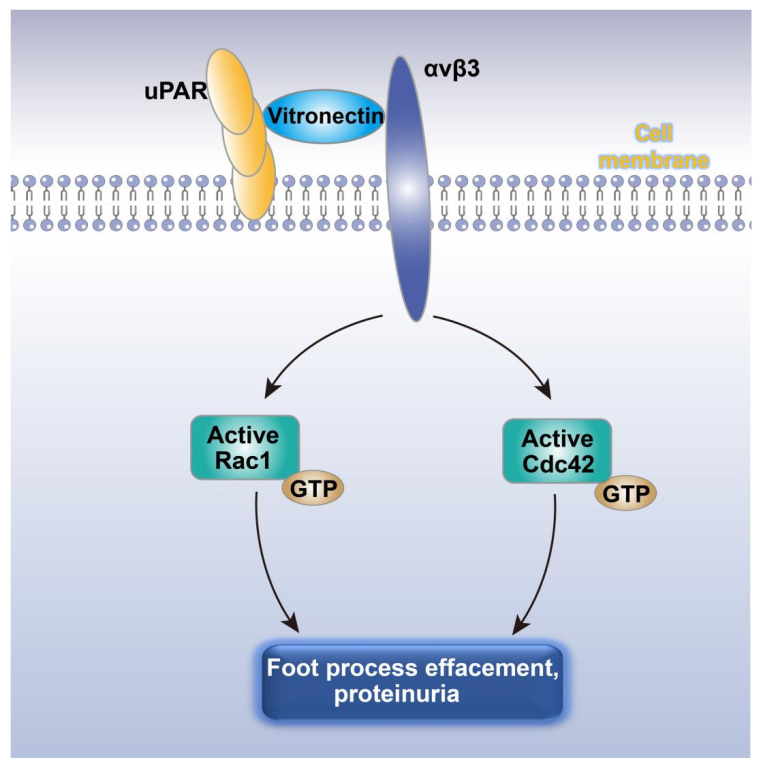
uPAR-αvβ3 pathway mediated foot process effacement and proteinuria through Rac1 and Cdc42. uPAR activates αvβ3 integrin through vitronectin in the lipid-rich domain of the plasma membrane of podocytes, mediates the increase in Cdc42 and Rac1 activity, and induces foot process fusion and proteinuria. uPRA, urokinase receptor.

Increased podocyte motility is the potential mechanism leading to foot process effacement [102]. Urokinase receptor (uPAR), a molecule associated with cell motility, was therefore identified as the target for researching the mechanism of proteinuria. The results showed that uPAR expression was significantly elevated in podocytes of patients with FSGS and diabetic nephropathy characterized by foot process effacement and proteinuria [119]. In rodent proteinuric diseases with elevated uPAR expression, β3 integrin was found to be activated by uPAR through vitronectin in the lipid-rich region of the podocyte plasma membrane and the inhibition of αvβ3 integrin not only reduced podocyte motility and proteinuria, but also decreased the elevation of Cdc42 and Rac1 activities (Figure 3) [119]. This indicated that the increased expression of uPAR in podocytes increased the activity of Cdc42 and Rac1 by activating αvβ3 integrin on the surface of podocytes, leading to increased podocyte motility, foot process effacement, and proteinuria [119].

In addition, ILK has been proven to regulate the actin cytoskeleton through a complex composed of ILK, PINCH, and parvin [120,121], among which parvin can bind to α-PIX (GEF); this complex can, therefore, regulate the Rac1 and Cdc42 activity [120].

Rac1 and Cdc42 are closely correlated with both angiogenesis and podocyte damage, indicating that compensatory pro-angiogenic factors produced by the kidneys of patients taking TKIs can theoretically act on diverse cells through Rac1 and Cdc42 at the same time, triggering different pathological phenotypes and clinical phenotypes. What is more, podocyte foot process effacement is reversible [94]. During the progression of FSGS, the threshold of podocyte apoptosis for glomerular injury irreversibility is 25‒40% of the initial podocyte number. If the stimuli are removed before this threshold, glomerular damage and proteinuria can be completely reversed [35,122]. Therefore, in the early stage of proteinuria caused by TKIs, it may be feasible to reverse proteinuria and podocyte injury by inhibiting the activities of Cdc42 and Rac1.

### 5.6. Molecules Involved in Angiogenesis and Podocyte Damage through Rac1 and Cdc42

When considering the role of compensatory pro-angiogenic factors induced by TKIs in angiogenesis, vascular barrier repair, and podocyte injury through Rac1 and Cdc42, Notch1 seems to be a candidate. In maintaining vascular barrier function, the transmembrane domain of Notch1 was revealed due to Notch1 ligand Dll4-dependent proteolytic activation, which was triggered by hemodynamic shear stress. This transmembrane domain catalyzed the formation of a receptor complex consisting of vascular endothelial cadherin, transmembrane protein tyrosine phosphatase LAR and Rac1 GEF Trio in the plasma membrane, which drove adhesion junction assembly and restored the barrier function by activating Rac1 [123]. In the axon growth and guidance of *Drosophila*, Notch also activated Rac through Trio [124]. On the contrary, in murine proteinuric nephropathy models, the podocyte-specific deletion of Sirt6 exacerbated podocyte injury and proteinuria [125]. In normal podocytes, Sirt6 inhibited the transcription of Notch1 and Notch4 by deacetylating H3K9, which protected podocytes from inflammation and apoptosis, and maintained the stability of the actin cytoskeleton [125]. More studies need to be conducted to further verify whether the downregulation of Notch1 expression reduces proteinuria through the decreased Rac1 activity. Furthermore, the change in Notch1 signaling after TKI treatment remains unclear. Solving these problems will contribute to clarifying the mechanism of proteinuria related to antiangiogenic therapy.

## 6. Podocyte Actin Cytoskeleton Regulatory Proteins

The molecules produced by damaged endothelial cells may also lead to podocyte injury through affecting proteins that regulate actin cytoskeleton, such as nephrin, α-actinin 4, synaptopodin, etc. [67].

The regulation of the podocyte actin cytoskeleton by synaptopodin has been described above. In normal podocytes, the administration of VEGFR2 TKI decreased the level of synaptopodin and increased the expression of TGF-β [74]. A recent study found that the glomerular injury induced by sorafenib (a multitarget TKI) was correlated with the decreased expression of synaptopodin, nephrin, podocin, and podoplanin [126].

Sorafenib may mediate proteinuria by blocking the downstream signaling pathway of nephrin through upregulating c-mip as well. Nephrin is a transmembrane protein expressed exclusively by the podocytes in the kidneys; it plays an important role in maintaining the integrity of the glomerular filtration barrier [67]. Under normal circumstances, the binding of activated Src family protein tyrosine kinase Fyn in the cell membrane to the cytoplasmic domain of nephrin promoted its phosphorylation. The phosphorylation of nephrin, on the one hand, triggered downstream phosphorylation events involving Nck, PAK-2, and N-WASP which promoted actin polymerization and cytoskeletal rearrangement; on the other hand, they activated Akt signaling pathway [127]. Sorafenib induced the overexpression of c-mip [28], which has been proven to reduce nephrin phosphorylation by directly binding to Fyn and preventing Fyn from interacting with nephrin and N-WASP [127]. Akt2 has been confirmed to have an inherent protective function in podocytes, and its knockout aggravated glomerulosclerosis and proteinuria by enhancing Rac1 activity [128]. Whether nephrin and AKT signaling pathway are involved in endothelial‒podocyte crosstalk in proteinuric nephropathies remains unclear. Activating the Akt signaling pathway may be feasible to protect podocytes and alleviate proteinuria, but the influence of its activation on the efficacy of TKIs due to its close relationship with the VEGFA/VEGFR2 signaling pathway cannot be ignored. This requires researchers to be very careful when using this method to treat proteinuria.

α-actinin 4 can be phosphorylated under the stimulation of EGF, which decreases the binding of α-actinin 4 with actin [129]. TGF-β can promote the phosphorylation of α-actinin 4 as well, leading to podocyte injury and proteinuria [130]. Hepatocyte growth factor (HGF) attenuated podocyte injury induced by puromycin aminonucleoside through inducing the restoration of α-actinin 4 and WT1 expression [131]. In NSCLC that is initially sensitive to VEGFR TKI, the development of resistance to treatment correlated with the increased expression of HGF and the activation of its receptor, c-MET. Dual inhibition of VEGFR/c-MET signaling delayed the resistance of NSCLC to VEGFR TKI [132]. It seems that tumor patients developing proteinuria after TKI treatment cannot benefit from HGF, but this may partly explain the predictive role of TKI-induced proteinuria on anticancer efficacy [133,134]—that is, patients with proteinuria may have low levels of HGF.

Other podocyte actin cytoskeleton regulatory proteins may also be involved in TKI-induced podocyte injury and proteinuria, but this requires further exploration.

## 7. Treatment

Many studies have been conducted on the treatment of proteinuric nephropathies, and a variety of agents have been proven to be good at reducing proteinuria [12,13,115,116,117,135,136,137,138], but most of them target the RAAS system and no drugs have been reported to be effective at improving the clinical outcome of proteinuric nephropathy induced by VEGFA‒VEGFR2 inhibitors.

Considering the reversibility and the role of podocyte injury in proteinuric nephropathies, the direct protection of podocytes during TKI therapy may prevent the occurrence of proteinuria. TRPC5 small-molecule inhibitor AC1903 has been confirmed to be effective at alleviating podocyte injury and proteinuria in ATIR-induced FSGS and hypertension-induced FSGS through disrupting the Rac1‒TRPC5 pathway in podocytes [109]. Another small molecule, Bis-T-23, showed the same effect in terms of reducing podocyte injury and proteinuria in diverse rodent models of proteinuric nephropathies [139]. Bis-T-23 protected podocytes by promoting the polymerization of dynamin, a large, multidomain GTPase that plays an important role in the regulation of the actin cytoskeleton [139]. Previous studies found that actin filaments, lipids, microtubules, and SH3-domain-containing proteins can promote dynamin oligomerizing into a higher-order structure [31] that can promote actin polymerization [139,140]. Podocyte-specific dynamin knockout presented massive proteinuria and podocyte foot process effacement [141]. These drugs alleviated proteinuria in various proteinuric kidney diseases by directly protecting podocytes and may be equally effective at relieving the proteinuria induced by TKIs.

Recent studies on *Fructus arctii*, a traditional herbal remedy used in treating diabetes and its complications, have found that its major component, arctigenin (ATG), reduces p65 NF-κB (also known as RelA)-mediated inflammation and Drebrin-1 (DBN1)-mediated podocyte injury by enhancing the activity of protein phosphatase 2 (PP2A) in podocytes, thereby protecting kidney function [142]. Previous studies have found that Rac1 can mediate renal inflammation, podocyte injury, and proteinuria by promoting the activation of NF-κB [94]. In fact, we have found the high expression of p65 NF-κB in mouse models of proteinuric renal disease caused by TKIs (unpublished data). This suggests that anti-inflammatory therapy may be helpful for alleviating the proteinuria induced by VEGFA‒VEGFR2 inhibitors.

No matter what methods are taken to address the proteinuria induced by VEGFA‒VEGFR2 inhibitors, the tumor progression of patients must be taken into account. Treatment regimens must be carefully screened to safely and effectively attenuate proteinuria without exacerbating tumor progression. The key to solve this problem is to thoroughly investigate the mechanism of proteinuric nephropathy induced by VEGFA‒VEGFR2 inhibitors.

## 8. Conclusions

In TKI-induced proteinuric kidney disease, podocyte injury may be a key factor, and endothelial cell damage may be the initial factor. TKI-induced endothelial cell damage leads to the compensatory expression of pro-angiogenic factors to repair vascular or promote angiogenesis. The compensatory pro-angiogenic factors result in podocyte injury by promoting the formation of abnormal endothelial‒podocyte crosstalk. Rac1 and Cdc42 play a crucial role in podocyte injury, angiogenesis, and vascular barrier repair, making them the key molecules for exploring the pro-angiogenic factors involved in TKI-induced proteinuric nephropathy.

## Figures and Tables

**Table 1:** The incidence of VEGF inhibitor-related proteinuria.

Drug	Class	Targets	Proteinuria (%)	References
Lenvatinib	TKI	VEGFRs, FGFR1–4, RET, KIT, PDGFRα	83	[4,19]
Regorafenib	TKI	VEGFRs, TIE2, KIT, RET	58	[3,19]
Apatinib	TKI	VEGFR2	47.7	[6,19]
Cediranib	TKI	VEGFRs, PDGFR, KIT,	37.8	[7,20]
Axitinib	TKI	VEGFRs, PDGFR, KIT	29.0	[8,21]
Linifanib	TKI	VEGFRs, PDGFR	27.3	[7,22]
Pazopanib	TKI	VEGFRs, PDGFR, KIT	13.5	[7,23]
Sorafenib	TKI	VEGFRs, PDGFRβ, RAF1, BRAF, KIT	11.6	[7,19]
Sunitinib	TKI	VEGFRs, PDGFR, KIT, RET, CSF1R, FLT3	11.1	[11,21]
Vandetanib	TKI	VEGFR2–3, RET, EGFR	10.0	[7,21]
Tivozanib	TKI	VEGFRs	9.6	[7,19]
Ramucirumab	mAb against VEGFR2	VEGFR2	20	[10,19]
Bevacizumab	mAb against VEGFA	VEGFA	21–62	[9]
